# Case report: a non-invasive approach to diagnosis and management of pericardial haemangioma

**DOI:** 10.1093/ehjcr/ytae545

**Published:** 2024-10-04

**Authors:** Franziska Adomat, Dominik A Steffen, Laurene Suter-Magpantay, André Linka, Lucas Weber

**Affiliations:** Department of Radiology and Nuclear Medicine, Cantonal Hospital Winterthur, Brauerstrasse 15, 8401, Winterthur, Switzerland; Department of Radiology and Nuclear Medicine, Cantonal Hospital Winterthur, Brauerstrasse 15, 8401, Winterthur, Switzerland; Department of Cardiology, Cantonal Hospital Winterthur, Brauerstrasse 15, 8401, Winterthur, Switzerland; Department of Cardiology, Cantonal Hospital Winterthur, Brauerstrasse 15, 8401, Winterthur, Switzerland; Department of Radiology and Nuclear Medicine, Cantonal Hospital Winterthur, Brauerstrasse 15, 8401, Winterthur, Switzerland

**Keywords:** Cardiac tumour, Pericardial haemangioma, Cardiac MRI, Non-invasive cardiac imaging, Case report

## Abstract

**Background:**

Pericardial haemangiomas represent a very rare subset of benign cardiac tumour in an unusual location, posing a diagnostic and clinical challenge. Historically, the definitive diagnosis was achieved through surgical resection or at biopsy. In recent years, multi-parametric cardiac magnetic resonance imaging (MRI) has proven to offer a non-invasive, biopsy-like approach to tumour characterization.

**Case summary:**

In our case, multimodality imaging was used to characterize a pericardial mass as a haemangioma discovered coincidentally with a brain glioma. Diagnostic certainty was substantially improved through utilization of successive post-contrast bright-blood imaging at cardiac MRI, demonstrating a characteristic enhancement pattern of haemangiomas in direct comparison to the blood pool. Conservative management and mid-term follow-up showed an uneventful clinical course and partial regression of the presumed pericardial haemangioma.

**Discussion:**

In the presence of typical features and application of individually tailored protocols, multimodality imaging can characterize cardiac tumours and guide patient management so that more invasive measures may be avoided. In our case of a suspected pericardial haemangioma, a conservative strategy was adopted with clinically uneventful course over a 2-year period. Whether this strategy can be applied to other patients with this rare tumour remains unclear, but the case report provides important information about the natural history of this entity and tissue characterization by cardiac MRI.

**Learning points**

- Pericardial haemangiomas are a very rare subset of benign cardiac tumours, historically diagnosed by invasive biopsy or surgery. Data regarding the natural history of the disease is scarce.
- Multimodality imaging with in-depth tissue characterization by multi-parametric cardiac magnetic resonance imaging can offer a high diagnostic certainty, without the need for invasive diagnostic procedures.
- Slightly delayed enhancement isointense to the blood pool in late venous and delayed phase without wash-out on T_1_ post-contrast bright-blood imaging are highly characteristic findings of haemangiomas.

## Introduction

Cardiac haemangiomas are a rare subset of benign cardiac tumours and pose both diagnostic and clinical challenges, especially when occurring in unusual locations like the pericardium. Historically, definitive diagnoses were established through surgical resection, at biopsy, or as an incidental finding at autopsy.^1^ Cardiac MRI has proven to offer a non-invasive, biopsy-like approach to the characterization of cardiac masses, improving diagnostic certainty when seen in combination with factors like prevalence, patient age, and tumour location.^2,3^ Diagnosing very rare cardiac tumours with atypical presentation is difficult and may necessitate multimodality imaging or invasive diagnostic techniques. Deciding between surgical or conservative management of pericardial haemangiomas is challenging given the scarcity of data on their natural history.

## Summary figure

**Figure:**
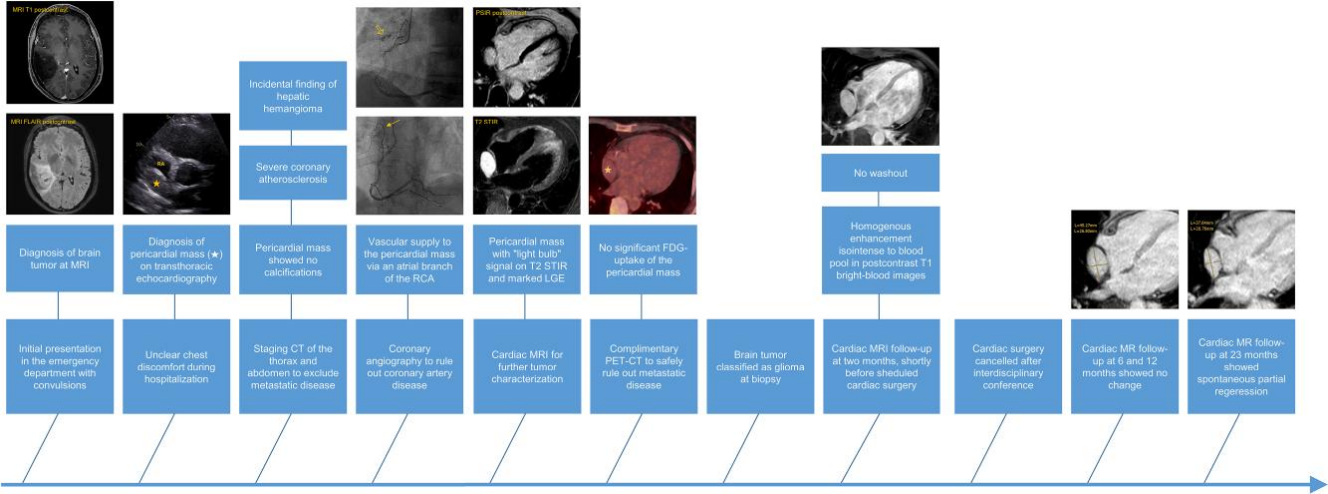


## Case history

**Figure 1:**
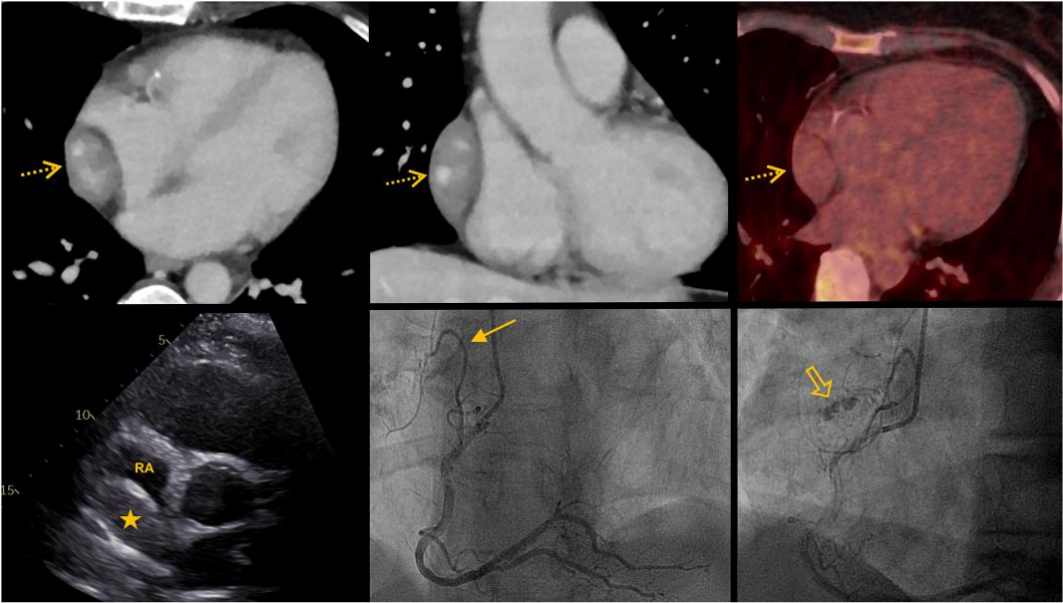
Multimodality imaging. Contrast-enhanced computed tomography in the portal venous phase in axial (*top* left) and coronal orientation (*top* middle) shows a well-demarcated, heterogeneously enhancing, ovoid mass adjacent to the right atrium (open arrows). Non-contrast positron emission tomography–computed tomography (*top* right) shows no significant fluorodeoxyglucose uptake in the pericardial mass (open arrow), instead remaining equivalent to the background blood pool activity. Transthoracic echocardiography in the parasternal short-axis view (*bottom* left) shows a well-demarcated, echogenic mass (star) adjacent to the roof of the right atrium. Coronary angiography showing blood supply to the tumour via an atrial branch of the right coronary artery (solid arrow; *bottom* middle) and ‘tumour blush’ of the mass (wide arrow; *bottom* right).

We present the case of a 48-year-old female patient with a previously bland medical history, who presented to the emergency department with convulsions, leading to the diagnosis of a brain tumour. During initial hospitalization, the patient experienced transient chest discomfort. In light of this, transthoracic echocardiography was performed, revealing a well-demarcated, ovoid, echogenic pericardial mass adjacent to the roof of the right atrium (*Figure 1*).

On suspicion of metastatic tumour disease, thoracoabdominal staging computed tomography (CT) was performed. This showed no further tumour manifestations, but an incidental finding of a hepatic haemangioma. The pericardial mass showed some enhancement centrally without any associated calcifications (*Figure 1*).

The presence of severe coronary atherosclerosis seen on staging CT prompted coronary angiography. This revealed a vascular supply to the pericardial mass via an atrial branch of the right coronary artery, while the tumour itself exhibited a ‘tumour blush’ (*Figure 1*). Coronary artery disease could be ruled out as a cause of chest discomfort.

**Figure 2:**
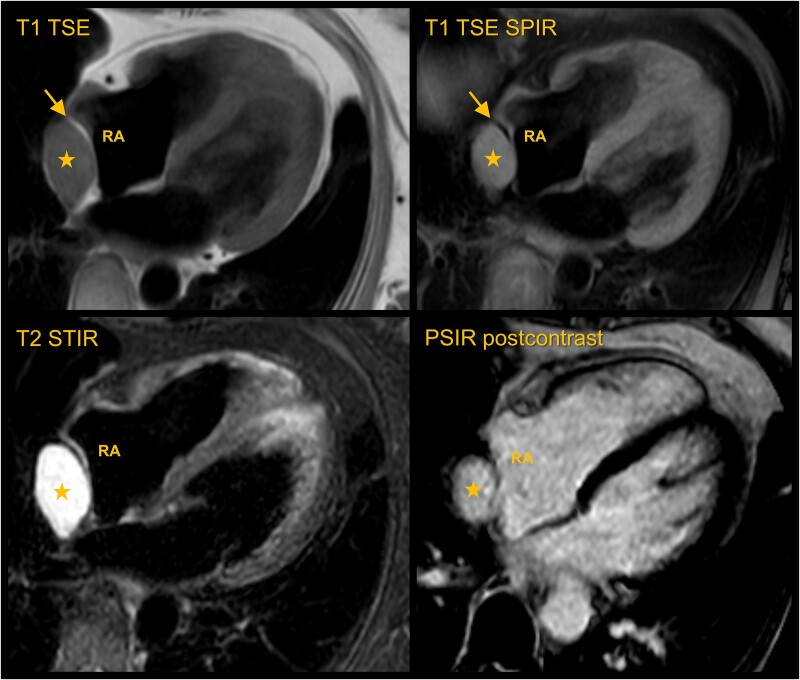
Multi-parametric cardiac magnetic resonance imaging. Conventional black-blood imaging shows a circumscribed tumour (star) clearly demarcated from the right atrial myocardium by a slim fatty lamella (arrow; *top* row), which demonstrates a signal drop in the fat-suppressed T_1_ turbo-spin-echo spectral pre-saturation with inversion recovery (*top* right). The tumour itself shows indifferent signal in T_1_ turbo-spin-echo black-blood (*top* left), no signal loss in T_1_ turbo-spin-echo spectral pre-saturation with inversion recovery black-blood, and markedly bright signal (‘light bulb’) in T_2_ short tau inversion recovery black-blood (*bottom* left). Post-contrast PSIR (*bottom* right) demonstrates homogeneous enhancement of the mass, isointense to the blood pool.

Cardiac MRI was performed for further tumour characterization. Conventional black-blood imaging revealed a circumscribed tumour in continuity with the pericardium, clearly demarcated from the right atrial myocardium by a slim fatty lamella (*Figure 2*), ruling out a myocardial origin. The tumour exhibited intermediate signal on T_1_ turbo-spin-echo (TSE) black-blood, no signal loss on fat-saturated T_1_ TSE SPIR black-blood, markedly bright signal (‘light bulb’) on T_2_ STIR black-blood (*Figure 2*) and marked late gadolinium enhancement (LGE). Avascular lesions, such as a thrombi or pericardial cysts, could be ruled out in the presence of contrast enhancement.^2^ A myxoma, the most common primary benign cardiac tumour, was deemed unlikely, as these are usually mobile intracavitary lesions with heterogeneous contrast uptake, though their myxoid stroma can present with ‘light bulb’ signal on T_2_.^4^ A lipoma could be ruled out due to the absence of macroscopic fat, which would have demonstrated marked signal loss on images with a fat saturation pre-pulse. The localization adjacent to the right atrium would be typical for an angiosarcoma, the most common malignant primary cardiac tumour, but no aggressive features such as infiltrative growth or necrosis were noted.^2,3^ While the pericardial mass exhibited typically benign characteristics and could represent a haemangioma, other entities such as a metastatic lesion, a low-grade sarcoma, or even a lymphoma could not be ruled out definitively at this point.

Based on clinical suspicion of metastatic disease, an interdisciplinary conference recommended a complementary positron emission tomography (PET)–CT, followed by surgical excision of the pericardial mass. Positron emission tomography–CT showed no metabolically active lesions in the chest or abdomen. The lack of significant fluorodeoxyglucose (FDG) uptake of the pericardial tumour (*Figure 1*) favoured a benign entity. Meanwhile, the brain tumour was histologically classified as a glioma, which is known to not metastasize systemically.

**Figure 3:**
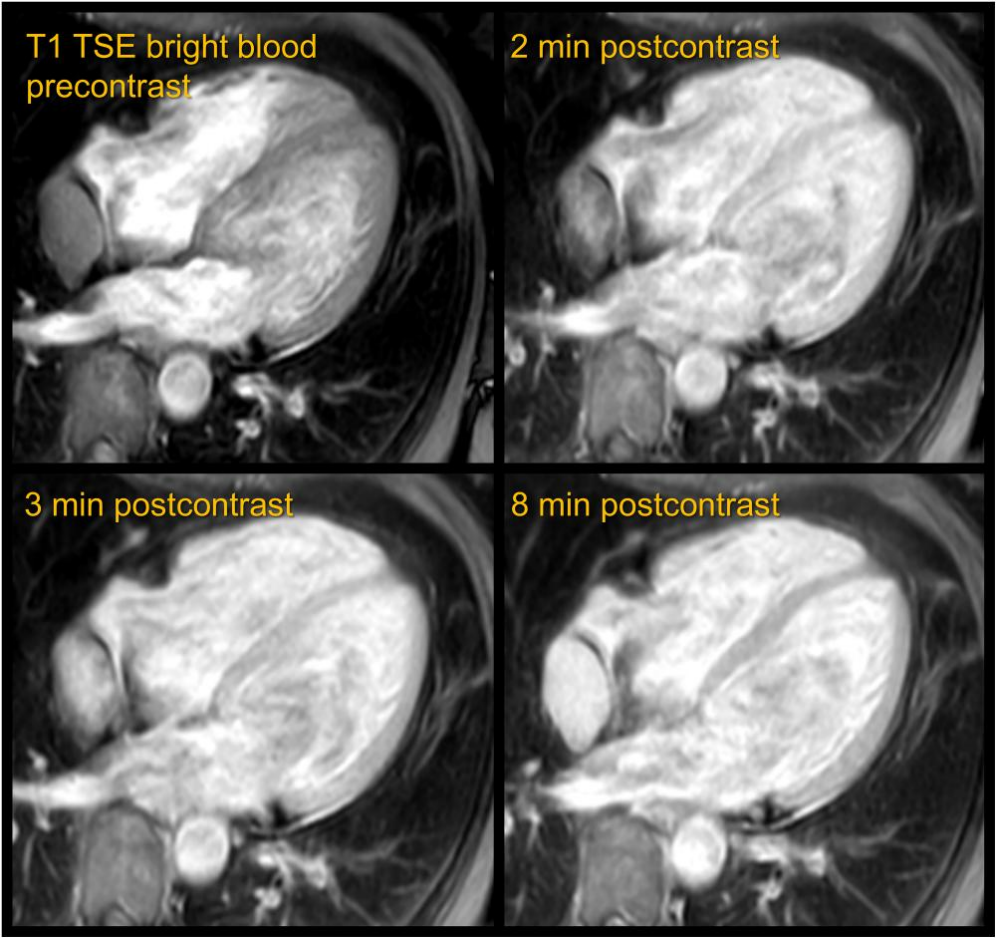
Cardiac magnetic resonance imaging with bright-blood post-contrast imaging. T_1_ turbo-spin-echo bright-blood images were acquired pre-contrast and successively after contrast administration, showing steadily progressing enhancement. Homogeneous enhancement of the tumour, isointense to blood pool, was achieved after 8 min, with persistence at 17 min (not shown). There was no evidence of wash-out, central necrosis, or thrombosis, thus proving the presence of a purely vascular lesion.

Shortly before scheduled cardiac surgery, the cardiac MRI was repeated with the primary purpose of ruling out short-term progression. The tumour remained consistent in size and showed identical imaging characteristics on black-blood sequences. In addition to the standard tumour protocol, dedicated sequential post-contrast T_1_ TSE bright-blood images were acquired on the suspicion of a haemangioma. Standard post-contrast black-blood images utilize a pre-pulse to suppress the signal from the blood pool and allow for better delineation of the myocardium. In contrast, bright-blood images do not suppress the signal from the blood pool, allowing for direct comparison of contrast enhancement to the blood pool. A typical finding of cavernous haemangiomas in other locations, such as the liver, is a centripetal filling pattern with peripheral rim or nodular enhancement in arterial phase, slightly delayed enhancement isointense to the blood pool in late venous and delayed phase without wash-out.^5^ Less commonly, atypical haemangiomas may exhibit arterial hyper-enhancement (flash-filling haemangiomas) or even a centrifugal enhancement pattern (inside-out haemangiomas). In this case, post-contrast T_1_ TSE bright-blood imaging demonstrated steadily progressing enhancement of the lesion from 1 to 5 min after contrast administration. Complete and homogeneous, blood pool isointense enhancement of the tumour was found after 8 min, persisting until the end of the examination at 17 min (*Figure 3*), highly characteristic for a haemangioma. A paraganglioma was deemed unlikely due to the homogenous nature of contrast enhancement with lack of contrast wash-in and wash-out.^6^ In this context, the markedly bright T_2_ signal should be interpreted as slow-flowing blood, rather than as a myxoid or cystic matrix.^7^

**Figure 4:**
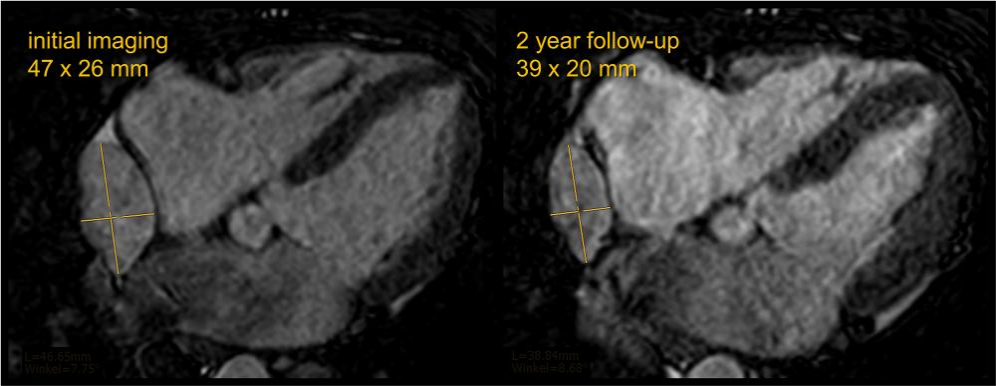
Follow-up by 3D balanced turbo field echo whole-heart imaging at cardiac magnetic resonance imaging, reconstructed in a four-chamber view. 3D balanced turbo field echo images obtained at the initial diagnostic time point (left) and at follow-up 2 years later (right) show a partial regression of this well-circumscribed lesion, which appears isointense to the blood pool.

At this point, repeated interdisciplinary consensus concluded that neither biopsy nor surgery was indicated, given high imaging certainty in regard to the tumour classification after repeated cardiac MRI, the absence of clinical deterioration, and concurrent diagnosis of glioma. Instead, a conservative management strategy was adopted. The presumed haemangioma remained stable in size at MRI follow-up after 6 and 12 months, while follow-up at 23 months showed spontaneous partial regression (*Figure 4*). No adverse cardiovascular events were noted at short interval clinical follow-up over 2 years.

## Discussion

Cardiac masses can be grouped into benign tumours, malignant tumours, and pseudotumours (*Table 1*). The distinction between these can be made on the basis of tissue characteristics, morphology, and location of the mass at multimodality imaging, under consideration of other factors such as patient age and prior medical history.^2,3,8,9^

**Table 1:** Overview of cardiac masses

Cardiac mass	Age at presentation	Most common location	General appearance	T_1_-weighted imaging	T_2_-weighted imaging	Contrast enhancement (LGE)
Benign						
Myxoma	Adults (30–60 years)	Left atrium (75%), with a predilection for the interatrial septum near the fossa ovalis	Well-defined, smooth, lobular or oval, often pedunculated and characteristically highly mobile; may contain regions of necrosis, fibrosis, haemorrhage, and calcifications	Isointense	Hyperintense	Heterogeneous
Papillary fibroelastoma	Adults	Valvular (left ventricular side of the mitral valve, aortic side of the aortic valve)	Usually small (<1.5 cm), mobile lesions with frond-like projections and a small pedicle; appears hypointense on cine imaging and may be associated with surrounding turbulence	Isointense	Usually hypointense, may appear partially hyperintense	No/minimal uptake
Lipoma	Any	Any (most commonly sub-epicardial)	Well-defined, homogeneous, encapsulated	Isointense to subcutaneous fat in all sequences	Isointense to subcutaneous fat in all sequences	No uptake
Fibroma	Children, young adults	Ventricles (LV > RV), usually arising from the interventricular septum	Homogeneous mass with central calcification	Hypo-/isointense	Homogenously hypointense	Hyper-enhancement, variable
Rhabdomyoma	Children (<1 year)	Ventricles (LV > RV; intramural)	Multiple (90%); small, well-circumscribed nodules or pedunculated mass; associated with tuberous sclerosis	Isointense	Hyperintense	No/minimal uptake
Haemangioma	Adults	Ventricles	Vascular neoplasms, typically solitary	Iso-/hyperintense	Hyperintense	Hyperenhancement, may be heterogeneous
Paraganglioma	Young adults	Left atrium	Infiltrative or circumscribed lesion with a broad base, may show regions of (central) necrosis	Iso-/hypointense, may be hyperintense due to haemorrhage	Hyperintense	Heterogeneous with peripheral rim enhancement
Malignant						
Angiosarcoma	Adults	Right atrium	Large, infiltrating mass with large regions of haemorrhage and necrosis	Heterogeneous	Heterogeneous	Heterogeneous, intralesional flow voids
Undifferentiated sarcoma	Adults (50–60 years)	Left atrium	Focal or infiltrative mass with regions of haemorrhage and necrosis	Isointense	Hyperintense	Heterogeneous, variable
Rhabdomyosarcoma	Children	Any (intramural, valvular)	Infiltrating mass, often multiple	Iso-/hypointense	Hyperintense	Homogeneous, variable in presence of central necrosis
Lymphoma	Adults	Right atrium	Infiltrating or nodular mass, frequently multiple	Iso-/hypointense	Iso-/hyperintense	No/minimal uptake
Metastasis	Any	Any	Variable appearance and location	Hypointense (hyperintense if melanoma)	Hyperintense (hypointense if melanoma)	Heterogeneous, variable
Pseudotumour						
Thrombus	Any	Left atrium, left atrial appendage	Mass of variable size and location. Frequently associated with atrial fibrillation and myocardial infarction.	Hypointense if chronic, hyperintense if acute or subacute	Hypointense if chronic or subacute, hyperintense if acute	No uptake (better delineated on early gadolinium enhancement sequences); organized thrombus may show some surface enhancement
Vegetations	Any	Valvular	Highly mobile, irregular masses causing valvular destruction and dysfunction			No uptake
Pericardial cyst	Any	Pericardium, usually located in the right pericardiophrenic angle	Congenital, circumscribed fluid-filled structures	Hypointense	Markedly hyperintense	No uptake

Note: Data are compiled based on references cited in the text.^2,3,8–10,13^

The most common cardiac masses are thrombi and metastatic lesions.^8^ Primary cardiac tumours are far less common, with an incidence of 0.002–0.3% and the majority representing benign entities (ca. 75%).^2,10^ Cardiac haemangiomas show a relative incidence of 3%, with pericardial haemangiomas comprising a location-specific subtype most commonly arising from the visceral layer of the pericardium, which can occur at any age, and be single or multiple.^11^ Rarely, they are associated with extrathoracic haemangiomas, as in our case with a finding of hepatic haemangioma.^1^ Usually, the diagnosis of pericardial haemangioma is made histopathologically, as they are either found incidentally at autopsy or resected for fear of (micro-)rupture with pericardial tamponade, or compression of adjoining structures.^1,11^ In women, increased risk of haemorrhage during pregnancy or hormone replacement therapy should be considered.^12^

Cardiac MRI can definitively characterize some cardiac lesions, such as cysts and lipomas, and reliably distinguish benign from malignant lesions. It is a very useful tool in narrowing the differential diagnosis of other cardiac masses, though tissue diagnosis will usually be required for a definitive diagnosis. Our case is one of the few in which a pericardial haemangioma was confidently diagnosed preoperatively by multimodality imaging and conservatively managed, expanding on the sparse data regarding the natural history of this entity. Echocardiography performed due to transient chest discomfort was able to confirm the presence of a mass. Invasive coronary angiography demonstrated characteristic ‘tumour blush’.^1,10,13,14^ Thoracoabdominal CT showed no macroscopic fat content or calcifications, and a concomitant liver haemangioma, while PET–CT was able to demonstrate a lack of significant FDG uptake, favouring a benign lesion. On cardiac MRI, the location of the lesion was definitively identified as pericardial, with in-depth tissue characterization demonstrating the typical findings of a vascular lesion with a contrast enhancement pattern highly characteristic of a haemangioma, the latter of which has been previously described in other cases of pericardial haemangioma.^5,7,13^ This was demonstrated in our case using a bright-blood sequential post-contrast T_1_ sequence not commonly employed in cardiac MRI protocols for tumour characterization.

In previously published cases of pericardial haemangioma, a large majority of patients underwent resection,^1^ which is associated with perioperative morbidity and mortality. Alternately minimally invasive therapy should be considered; isolated case reports describe treatment of cardiac and pericardial haemangiomas via embolization, coiling, or stent graft placement.^14,15^ In contrast to invasive surgery, the risk of relevant complications caused by this benign tumour seem to be quite small, which is why we considered a watchful waiting strategy to be the most sensible course of action in our patient with concomitant brain glioma. Mid-term clinical and imaging follow-up showed no adverse cardiovascular events and partial regression of the pericardial mass. It should be noted that the cause of initial chest discomfort remained unclear and did not recur; however, after exclusion of coronary artery disease, it was thought to have been associated with the preceding epileptic seizure or as musculoskeletal discomfort.^11,16^ The lack of biopsy with histological correlation of the lesion, which serves as the gold standard for tumour classification, remains a limitation of this case report; however, Zeina *et al*.^7^ and Omura *et al*.^13^ describe similar findings at multimodality imaging in case reports of histologically confirmed pericardial haemangiomas.

Multimodality imaging with individually tailored multi-parametric cardiac MRI is of significant value in cardiac tumour characterization and often guides patient management. In our case, biopsy with histopathological correlation was deemed unnecessary and a conservative strategy was employed, with an uneventful clinical course over 2 years. Larger case series or studies are needed to determine if this strategy can be safely utilized in other asymptomatic patients with pericardial haemangiomas.

## Lead author biography


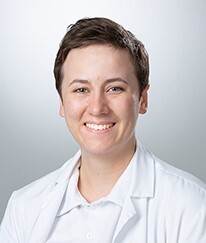


Franziska Adomat currently works at the Department of Radiology and Nuclear Medicine at St. Claraspital in Basel and the Cantonal Hospital of Winterthur, Switzerland, where she previously worked as a fellow for cardiovascular imaging. Her passion lies in abdominal and cardiac imaging.

**Consent:** The authors confirm that written consent for submission and publication of this case report has been obtained from the patient, in keeping with the COPE guidelines.

**Funding:** None declared.

## Data Availability

No data was generated or analysed during the study.
